# Comparison of tendon and muscle belly vibratory stimulation in the treatment of post-stroke upper extremity spasticity: a retrospective observational pilot study

**DOI:** 10.1038/s41598-024-54815-1

**Published:** 2024-02-20

**Authors:** Kenta Takeuchi, Takashi Takebayashi, Daiki Hanioka, Yuho Okita, Shinichi Shimada

**Affiliations:** 1 Department of Rehabilitation, Itami Kousei Neurosurgical Hospital, 1-300-1, Nishino, Itami, Hyogo Japan; 2 Department of Occupational Therapy, Graduate School of Comprehensive Rehabilitation, Osaka Prefecture University, Osaka, Japan; 3 Department of Occupational Therapy, Graduate School of Comprehensive Rehabilitation, Osaka Metropolitan University, Osaka, Japan; 4 Department of Rehabilitation, Tsukazaki Hospital, Himeji, Japan; 5 Soaring Health Sport, Wellness & Community Centre, Melbourne, Australia; 6 Department of Neurosurgery, Itami Kousei Neurosurgical Hospital, Itami, Japan

**Keywords:** Neurological disorders, Rehabilitation

## Abstract

Previous studies have reported the effects of vibratory stimulation (VS) therapy in reducing upper extremity spasticity after stroke. However, the effective location of the VS in patients with stroke remains unclear. This study aimed to determine the VS location that is most effective in reducing post-stroke finger and wrist flexor spasticity. We enrolled 27 consecutive patients with stroke and upper extremity spasticity in this retrospective observational study. The participants received stretching, tendon vibration, and muscle belly vibration for 5 min over a period of 3 days. To evaluate spasticity, we assessed the Modified Ashworth Scale score before and immediately after each treatment and immediately after voluntary finger flexion. Participants who received tendon vibration showed greater improvement in flexor tone in the fingers than participants who received stretching and muscle belly vibration (*P* < 0.05 and < 0.001, respectively). Participants who underwent VS showed no significant improvement in the wrist flexor tone compared to those who underwent stretching. Our results suggest that the tendon may be the most effective location for treating spasticity of the finger flexor muscles and that VS may not significantly improve spasticity of the wrist flexors more than stretching.

## Introduction

Spasticity is a movement disorder characterized by a velocity-dependent increase in muscle tone, with exacerbated tendon reflexes^1^. Upper extremity spasticity occurs in approximately 35% of patients within 6 months of stroke onset^2^. Severe spasticity is more common in the upper than in the lower extremities^2^. Stroke patients may experience joint contractures, muscle pain, and limitations in their daily activities due to spasticity. This spasticity can also become a barrier to improving upper extremity function^3,4^. This highlights the importance of treating spasticity to facilitate the improvement of upper extremity hemiparesis.

Although botulinum toxin injection^5–8^ and intrathecal baclofen therapy^9^ are well-tolerated and effective treatments for spasticity after stroke, these therapies require special clinical skills and are invasive procedures that may cause pain. Moreover, these therapies are usually not cost-effective^10^ due to their high cost. In contrast, stretching^11^, extracorporeal shock wave therapy^12,13^, neuromuscular electrical stimulation^14^, and vibratory stimulation (VS)^15–17^ serve as non-pharmacological and non-invasive alternatives for the treatment of spasticity.

The American Heart Association guidelines recommend that VS be considered a non-invasive and effective treatment for reducing spasticity^18^. Three randomized controlled trials reported that local muscle VS may be a useful tool with anti-spastic effects when applied directly to the spastic muscles of the hemiplegic upper extremity after stroke^15–17^. Of these studies, Noma et al.^15^ implemented VS on the tendon and Costantino et al.^16^ implemented VS on the muscle belly; both studies reported improvement in muscle tone as measured by the Modified Ashworth Scale (MAS). However, no previous studies have compared the effects of VS on the muscle belly and tendon on upper extremity spasticity in patients with stroke. Owing to a lack of knowledge, no consensus has been reached as to whether the muscle belly is a more effective location than the tendon in VS therapy for spasticity. A recent systematic review of the effectiveness of VS for spasticity in patients with stroke also suggested that treatment effectiveness may vary depending on the target muscles and the degree of spasticity^19^.

This study aimed to determine whether VS of the tendon or muscle belly is more effective in reducing spasticity of the finger and wrist flexors in patients following stroke. Investigating the most effective location may enhance the effectiveness of VS therapy for spasticity in patients with stroke.

## Methods

### Study design and research participants

**Figure 1:**
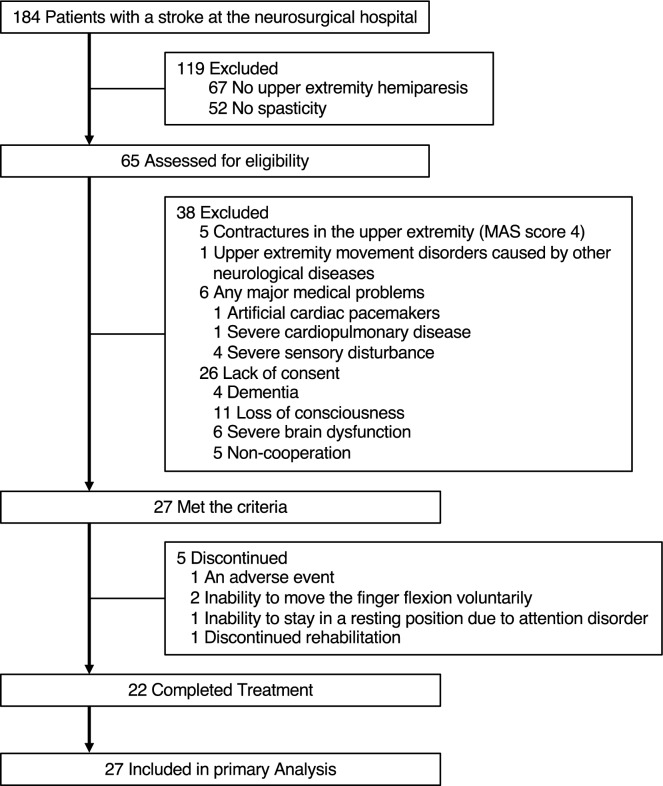
Participant flow in the present study.

This retrospective observational study was conducted at our hospital. We recruited 27 consecutive patients with stroke who met the following criteria (Fig. 1): upper extremity hemiparesis, abnormal muscle tone of the affected wrist or finger flexors (MAS score 1–3); age > 20 years, and providing informed consent between November 2018 and March 2019.

The exclusion criteria were as follows: contractures in the upper extremity (MAS score 4), upper extremity movement disorders caused by other neurological diseases, and any major medical problems determined by the physician (artificial cardiac pacemakers, severe cardiopulmonary disease, or severe sensory disturbance).

The study was approved by the nonprofit organization MINS Institutional Review Board (acknowledgment number: 180229), and was designed according to the 1975 Declaration of Helsinki, as revised in 1983. The trial was registered in the University Hospital Medical Information Network Clinical Trial Registry (UMIN000043457), which is a public trial registry.

### Procedure

**Figure 2:**
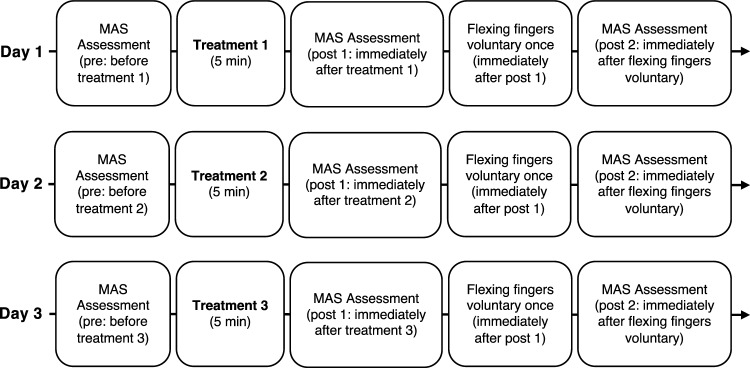
Study design time flow. The order of the three treatments was randomized for each participant. Three treatments consisted of stretching for the wrist and finger flexor muscles, vibration stimulation therapy on the tendon of the wrist and finger flexor muscles, and vibration stimulation therapy on the muscle belly of the wrist and finger flexor muscles. MAS: Modified Ashworth Scale.

Figure 2 shows the typical procedure. The participants received three treatments once a day for 3 days. The order of treatment was randomized for each participant by the author. Occupational Therapist in charge of patient’s treatment performed the treatments and evaluations. The authors created a manual for the evaluation and treatment procedures based on previous studies^15,20^. We handed it out to the occupational therapists responsible for the evaluation and treatments. All occupational therapists underwent instruction in the methodology for assessing muscle tone through the Modified Ashworth Scale (MAS) and received training in three distinct therapeutic modalities under the author's guidance during practical skills development. The MAS scores were recorded three times for each treatment: before treatment (pre), immediately after treatment (post 1), and immediately after voluntary finger flexion (post 2).

Participants flexed their fingers voluntarily immediately after recording the post 1 MAS scores, which were recorded again (post 2) to assess the lasting effect of reducing spasticity.

This study is not blinded in its evaluation, treatment, or analysis.

### Interventions

The participants received the following three treatments: (1) stretching of the wrist and finger flexors, (2) VS therapy for the tendon of the wrist and finger flexors, and (3) VS therapy for the muscle belly of the wrist and finger flexors. The participants received each treatment for 5 min. During each treatment, the participants lay in the supine position and were asked to relax their muscles, as muscle contractions can interfere with the effects of the VS. If they could not receive the interventions in the supine position, they received the treatments in the sitting position.

**Figure 3:**
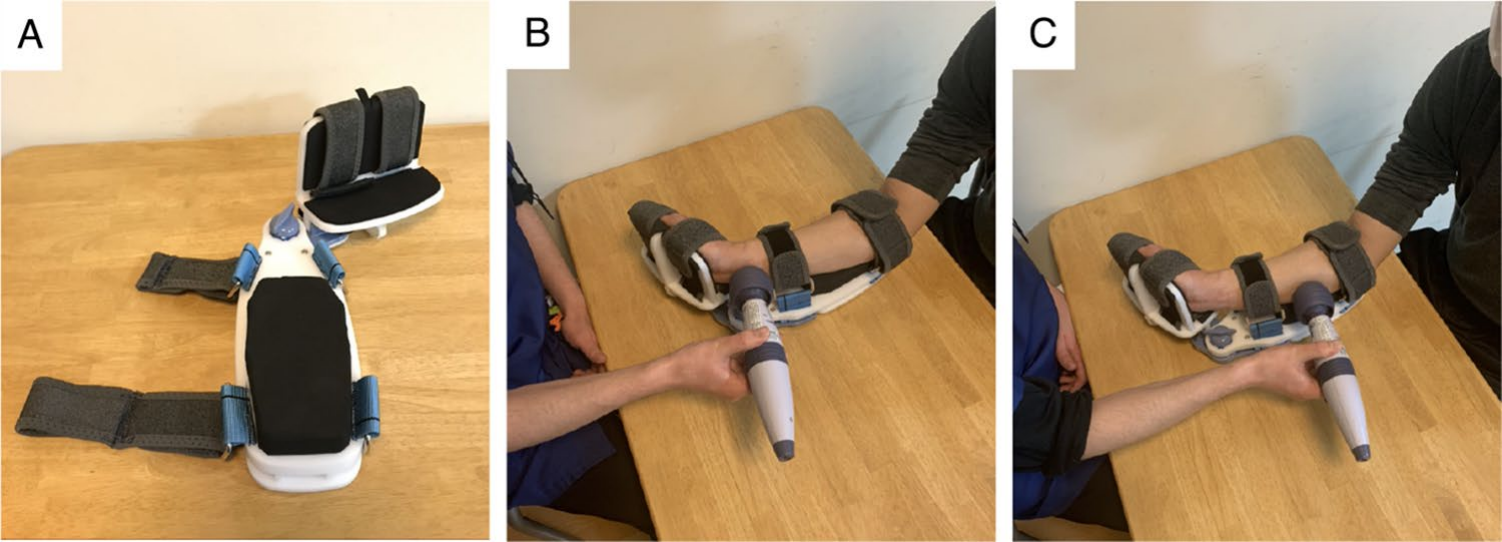
The device used to fix the upper extremity and apply vibratory stimulation. (**A**) The Wrist and finger joints placed in the maximal extension position. (**B**) Vibratory stimulation on the tendon of the wrist and finger flexor muscles. (**C**) Vibratory stimulation on the muscle belly.

In each treatment, the participant’s arm was placed in the maximum extension position using a specific device (Fig. 3A) to suppress the initial intense contraction by VS. The device had a movable wrist joint section that could be adjusted to fix the wrist joint in the maximum extension position. The device and treatment methods used were based on a previous study^15^. The device was developed by Teijin Pharma Ltd, Tokyo, Japan.

VS was applied to the tendon or muscle belly of the wrist and finger flexors using a vibration massager with spherical rubber and a vinyl-covered head (diameter: 5 cm) (Thrive MD-01; Thrive Co. Ltd., Osaka, Japan) (Fig. 3B,C). The vibration frequency was set to 91 Hz at an amplitude of 1.0 mm.

### Measurement of muscle tone

The MAS^20^ was used to individually evaluate the spasticity of the wrist and finger flexors, and the scales rated the resistance perceived when moving an extremity passively about a joint in six grades (0, 1, 1+, 2, 3, and 4). A score of 0 indicates normal muscle tone, and 4 indicates rigid flexion or extension. MAS has mainly been used in previous studies to evaluate the spasticity of the biceps brachii, wrist flexors, and finger flexors^20^. For data analysis, MAS scores (0, 1, 1+, 2, 3, and 4) were assigned numerical values called “calculated MAS scores” (0, 1, 2, 3, 4, and 5, respectively)^21^.

We placed the joint in a maximally flexed position and moved it to a position of maximal extension for one second^20^.

The response rate was used to evaluate the response of spasticity to each treatment. This was defined as the proportion of participants with at least a 1-point improvement from baseline (pre) on the MAS.

### Statistical analysis

No statistical sample size calculations were conducted. However, we conducted post-hoc power and effect size analyses using the results for the 27 participants in this study in GPower version 3.1. We calculated post-hoc power and effect size from the sum of the squares calculated in the analysis of variance.

This study included data from participants who did not complete the entire study process in the data analysis. Non-parametric statistics were used for the analyses because not all data met the normality criterion. This study did not compare patient characteristics between treatments because the three different treatments were administered to the same subjects. The effects of the interventions over time on the MAS scores were evaluated using 2-way repeated measures analysis of variance (ANOVA) with a post-hoc Bonferroni-corrected Wilcoxon test (number of comparisons = 3). Between-group differences in MAS scores were analyzed using the Kruskal–Wallis test and Bonferroni-corrected Wilcoxon test (number of comparisons = 3). To compare response rates between interventions, McNemar’s test was conducted. The threshold for significance was set at *P *< 0.05. All statistical analyses were performed using JMP version 17.0 software (SAS Institute Japan, Tokyo, Japan).

## Results

**Figure 4:**
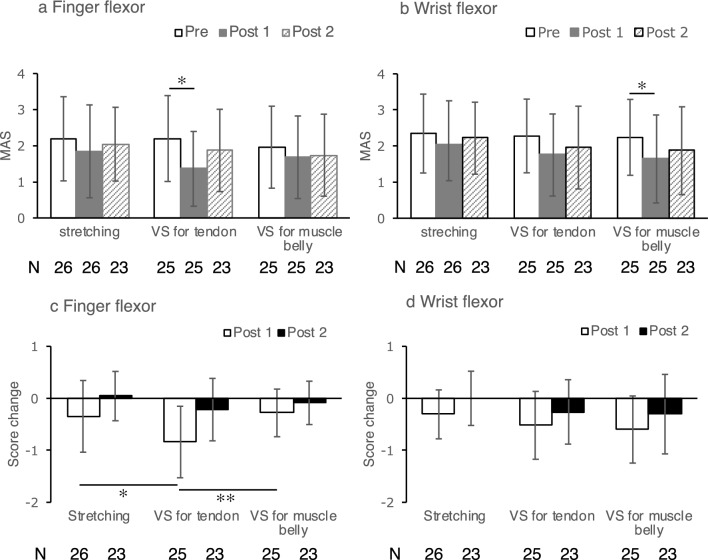
Modified Ashworth Scale (MAS) score changes. MAS score changes for the finger (**a**,**c**) and the wrist flexors (**b**,**d**) following the three interventions are shown. Values are presented as mean and standard deviations. Post 1 corresponds to 5 min after vibratory stimulation. Post 2 corresponds to the moment after one voluntary flexion movement after Post 1 assessment. N indicates the number of patients analyzed in each bar. Significant differences among three interventions are indicated at *P < 0.05 and **P < 0.01. VS: vibratory stimulation.

**Figure 5:**
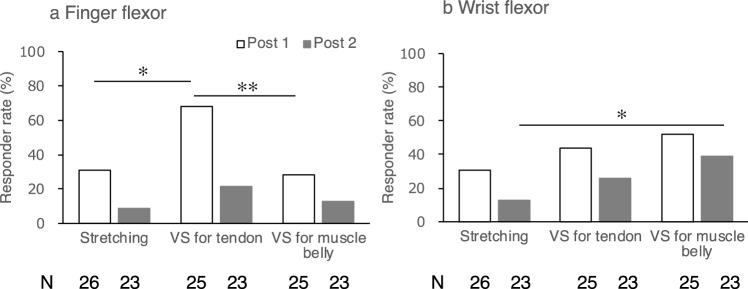
Response rate of finger (**a**) and wrist flexors (**b**) for the three interventions are shown. Post 1 corresponds to 5 min after vibratory stimulation. Post 2 corresponds to the moment after one voluntary flexion movement after Post 1 assessment. N indicates the number of patients analyzed in each bar. Significant differences among three interventions are indicated at *P < 0.05 and **P < 0.01. VS: Vibratory stimulation.

Table 1 provides participants’ demographic information. The number of participants included in the analysis for each treatment is shown at the bottom of Figs. 4 and 5. One participant presented with blushing, a hot feeling, and swelling as adverse events after VS of the muscle belly. However, the symptoms improved the following day. Five participants received partial treatment because of an adverse event (N = 1), inability to move the finger flexion voluntarily (N = 2), inability to stay in a resting position due to attention disorder (N = 1), and discontinued rehabilitation (N = 1).

**Table 1:** Participant demographics.

Age in years, mean (SD), range	61.3 (12.4), 40–84
Sex (male), n (%)	16 (59.3)
Dominant hand (right), n (%)	27 (100)
Side of hemiparesis (right), n (%)	12 (44.4)
Stroke type, n (%)	
Ischemic	10 (37.0%)
Cerebral hemorrhage	17 (63.0%)
Subarachnoid hemorrhage	1 (4%)^a^
History of stroke, n (%)	
Initial	23 (85%)
Recurrent	4 (15%)
Time since stroke in days, median (IQR)	99 (69–168)
Brunnstrom recovery stage of upper extremity	
II	8
III	11
IV	8
Brunnstrom recovery stage of fingers	
II	8
III	11
IV	8

^a^One patient had both cerebral hemorrhage and subarachnoid hemorrhage.

Table 2 and Fig. 4 show the changes in MAS scores for the finger and wrist flexors after each intervention. The repeated-measures ANOVA showed significant differences over time in the MAS scores of the finger flexors (*P* < 0.05) and wrist flexors (*P* < 0.05).

**Table 2:** Modified Ashworth Scale at preintervention, postintervention 1, postintervention 2.

	Preintervention	n	Postintervention 1	n	Change from pre	Postintervention 2	n	Change from pre
	Mean (SD)		Mean (SD)		Mean (SD)	Mean (SD)		Mean (SD)
Finger flexor								
Stretching	2.19 (1.17)	26	1.85 (1.29)	26	− 0.35 (0.69)	2.04 (1.02)	23	0.04 (0.47)
VS for tendon	2.20 (1.19)	25	1.36 (1.04)	25	− 0.84 (0.69)	1.87 (1.14)	23	− 0.22 (0.60)
VS for muscle belly	1.96 (1.14)	25	1.68 (1.14)	25	− 0.28 (0.46)	1.74 (1.14)	23	− 0.09 (0.42)
Wrist flexor								
Stretching	2.35 (1.09)	26	2.04 (1.22)	26	− 0.31 (0.47)	2.22 (1.00)	23	0.00 (0.52)
VS for tendon	2.28 (1.02)	25	1.76 (1.13)	25	− 0.52 (0.65)	1.96 (1.15)	23	− 0.26 (0.62)
VS for muscle belly	2.24 (1.05)	25	1.64 (1.22)	25	− 0.60 (0.65)	1.87 (1.22)	23	− 0.30 (0.76)

Postintervention 1: immediately after treatment 1.

Postintervention 2: immediately after flexing fingers voluntary.

The post-hoc test revealed a decrease in the MAS score for the finger flexors after tendon vibration (pre vs post 1, *P* < 0.01) and the wrist flexors after muscle belly vibration (pre vs post 1, *P* < 0.05).

The Kruskal–Wallis test showed significant differences over time among the three treatments in the MAS scores of finger flexors (post 1, *P* < 0.01). Conversely, the Kruskal–Wallis test showed no significant differences over time among the three treatments in the MAS scores of the wrist flexors.

Participants who received VS on the tendon showed greater improvement in MAS scores for the flexor tone of the fingers than participants who received stretching (post 1, P < 0.05) or VS on the muscle belly (post 1, *P* < 0.001).

We also analyzed the response rate to clarify the effects of each intervention on spasticity (Fig. 5). In the treatment of finger flexor spasticity, McNemar’s test showed that the response rate of VS on the tendon (68%) was significantly higher than that of stretching (31%) and VS on the muscle belly (28%) (*P* = 0.012 and *P* = 0.004, respectively). These results suggest that VS on the tendon may be more effective in reducing finger flexor spasticity. In the treatment of wrist flexor spasticity, although there was no significant difference at post 1, the response rate of VS on the muscle belly (39%) was higher than that of stretching (13%) at post 2 (*P* = 0.031). These results suggest that muscle belly vibration may be more effective in reducing the spasticity of the wrist flexors and may provide a lasting effect on reducing spasticity after one voluntary finger flexion movement.

A post-hoc analysis confirmed sufficient power (1−β = 0.99) and effect size (0.23) in the treatment of finger flexor spasticity and power (0.99) and effect size (0.21) in the treatment of wrist flexor spasticity.

## Discussion

This study was conducted using retrospective observational methods to confirm whether VS of the tendon or muscle belly was more effective in reducing the spasticity of the finger and wrist flexors in patients following stroke. Previous studies^15–17^ reported that VS on the tendon and muscle belly reduces spasticity after stroke; however, the most effective location for reducing spasticity remains to be clarified. To the best of our knowledge, the present study is the first to demonstrate the most effective VS location for reducing finger and wrist flexor muscle spasticity. Our findings suggest that participants who underwent VS on the tendon showed greater improvement in MAS scores in the flexor tone of the fingers than those who underwent stretching and VS on the muscle belly. In the treatment of finger flexor spasticity, the tendon VS response rate was significantly higher than that of stretching and muscle belly VS at post 1. Participants who underwent VS showed no greater improvement in MAS scores in the flexor tone of the wrist than those who underwent stretching. In the treatment of wrist flexor spasticity, the response rate of muscle belly VS was significantly higher than that of stretching at post 2.

This study showed that VS on the tendon was more effective in reducing finger flexor spasticity than on the muscle belly. This may be biologically plausible because tendon VS influences the Golgi tendon organ, which can induce Ib afferent inhibition, leading to a decrease in the tonus of the stimulated muscle^22^ in a similar way to stretching. Second, the flexor digitorum superficialis muscle, which is involved in the flexion of the four fingers, is anatomically deeper than the palmaris longus and flexor carpi radialis muscles, which are involved in wrist flexion. VS on the proximal forearm may therefore not effectively stimulate the flexor digitorum superficialis muscle. However, VS on the distal forearm may more effectively stimulate the tendon of the flexor digitorum superficialis muscle, thereby spreading the effects of the VS from the tendon to the muscle belly. Thus, it is plausible that VS on the tendon in the distal forearm may affect the flexor digitorum superficialis muscle, resulting in reduced flexor tone in the fingers.

VS on the tendon and muscle belly of the wrist flexors showed no significant effect compared with stretching. A previous randomized controlled study^16^ showed the effects of VS on the muscle belly for wrist spasticity, but the results of that and our study cannot be directly compared since the previous study^16^ provided 30 min per session, three times per week, for a total of 4 weeks of treatment. We could not replicate the effects of stretching and VS on the tendon for wrist spasticity seen in another randomized controlled study^15^. We speculate that the device used to fix the wrist joint in the maximum extension position may not have provided sufficient stretching. In the treatment of wrist flexor spasticity, the response rate of VS on the muscle belly was significantly higher than that of stretching at post 2. This result implies that VS of the muscle belly may be effective in reducing spasticity of the wrist flexors.

In this study, one patient reported blushing, a hot feeling, and swelling as adverse events associated with VS. These adverse events were attributed to pressing the head of the vibrator hard against the participant’s skin. Although one of the authors trained all therapists in the use of the vibrator and application of VS, maintaining consistent pressure of the vibrator head on the skin was difficult for all therapists. In conclusion, it is advisable to use a stimulation device, such as the DAVS^15^, to equalize the pressure of the vibrator head on the skin among therapists in a clinical setting.

This study has several limitations. Thus, our findings should be interpreted with caution. First, this was a non-randomized, retrospective, observational pilot study. The evaluators and therapists were not blinded; therefore, our results have several potential biases. Prospective randomized trials are needed to compare tendon, muscle belly VS, and a control group without VS and to clarify the efficacy of VS in patients with stroke and spasticity. Second, this study included a small number of participants (N = 27) and did not determine the optimal sample size to ensure adequate power. Therefore, it is not sufficient to generalize the effectiveness of VS for spasticity. However, despite the limited number of participants, a clear pattern emerged, suggesting that VS on the tendon had a positive effect on the spasticity of the finger flexor muscles. Third, as this was an exploratory pilot study, we included in our analysis some participants who did not complete the three treatments and had missing data. This may have led to the inclusion of potential bias. Thus, future studies should have an experimental design that does not produce missing data, and procedures to correct for missing data. Forth, the inclusion criterion was extensive. The time since the onset of stroke was not included in this study. However, we believe it may have had little impact on the results considering participants were not patients with acute stroke; thus, their short-term differences in prognosis were limited (Table 1) and the intervention period was short. The inclusion criteria did not include age or the severity of motor impairment; thus, future studies are needed to include these factors in the inclusion criteria. Fifth, this study relied only on the MAS as a subjective clinical measurement to evaluate spasticity; thus, future studies are needed to evaluate motor function and activity limitations for upper extremity rehabilitation, and neurophysiological changes using F-wave measurements^15^ as an objective measurement. A previous randomized controlled study^15^ showed that VS on the tendon significantly decreased F-wave parameters and that F-wave parameters were significantly correlated with MAS scores. A similar phenomenon may have occurred in our study. In contrast, no previous studies have demonstrated the effects of VS on the muscle belly on spasticity using electromyographic assessments. Future studies are needed to examine the effects of VS on the tendon and muscle belly using neurophysiological assessments, because they may differ in terms of physiological mechanisms. Sixth, in this study, there was a lack of uniformity in postural alignment among the participants during the assessment and treatment of spasticity. Postural differences could have affected the muscle tone in the upper extremities. Seventh, a previous study showed that patients with spasticity can experience a level of fibrotic change in the spastic muscle^23^. Thus, future studies are needed to use ultrasound to measure the structural features of the muscles and assess their impact on outcomes. Finally, it is uncertain how long the spasticity reduction lasts after treatment. Future studies will require long-term follow-up.

## Conclusion

Although it is too early to draw statistically significant conclusions, two patterns seem to be emerging: tendon vibration significantly improved the MAS scores of the finger flexors compared with stretching and muscle belly VS. Participants who underwent VS showed no significant improvement in the wrist flexor tone compared to those who underwent stretching. These findings suggest that the tendon may be the most effective location for treating spasticity of the finger flexor muscles and that VS may not significantly improve spasticity of the wrist flexors more than stretching. As our study clearly has some limitations, our findings might not be generalizable to a clinical setting. Further studies with high-quality methodology and large sample sizes are needed to determine the most effective location to enhance the effectiveness of VS therapy for spasticity in patients with stroke.

## Data Availability

The datasets used during the current study are available from the corresponding author on reasonable request.
